# Synthetic lincosamides iboxamycin and cresomycin are active against ocular multidrug-resistant methicillin-resistant *Staphylococcus aureus* carrying *erm* genes

**DOI:** 10.1016/j.jgar.2024.09.001

**Published:** 2024-09-16

**Authors:** Camille André, Kelvin J.Y. Wu, Andrew G. Myers, Paulo J.M. Bispo

**Affiliations:** a Department of Ophthalmology, Massachusetts Eye and Ear Infirmary, Harvard Medical School, Boston MA, USA; b Department of Chemistry and Chemical Biology, Harvard University, Cambridge, MA 02138, USA

**Keywords:** Multidrug resistance, MRSA, *Erm* genes, New antibiotics

## Abstract

**Objective:**

Antimicrobial resistance is a global pandemic that poses a major threat to vision health as ocular bacteria, especially methicillin-resistant *Staphylococcus aureus* (MRSA), are becoming increasingly resistant to first-line therapies. Here we evaluated the antimicrobial activity of new synthetic lincosamides in comparison to currently used antibiotics against clinical ocular MRSA isolates.

**Methods:**

Antimicrobial susceptibility testing was performed by broth microdilution for two novel synthetic lincosamides (iboxamycin and cresomycin) and eight comparator antibiotics against a collection of 50 genomically characterised ocular MRSA isolates, including isolates harbouring *erm* genes (*n* = 25).

**Results:**

Both drugs were active against widespread MRSA clonal complexes CC8 and CC5. The MIC_50_ and MIC_90_ of iboxamycin were 0.06 and 2 mg/L, respectively. Cresomycin (MIC_50_ = 0.06 mg/L) also displayed good activity with an *in vitro* potency four-fold higher (MIC_90_ = 0.5 mg/L) than iboxamycin. In isolates harbouring *erm* genes, MIC_90_ were *>*16, 2, and 0.5 mg/L for clindamycin, iboxamycin, and cresomycin, respectively. The *in vitro* potencies of iboxamycin and cresomycin were similar or higher than that of comparator agents and were not impacted by multidrug-resistance phenotypes or by the presence of *erm* genes when compared with clindamycin.

**Conclusions:**

Our results demonstrate that iboxamycin and cresomycin display potent *in vitro* activity against ocular MRSA isolates, including multidrug-resistant isolates harbouring *erm* genes.

## 1. Introduction

Bacteria are the most common etiologies of eye infections, with Gram-positive organisms such as *Staphylococcus aureus* among the leading causes [1]. *S. aureus* is notable for its ability to acquire and express various antimicrobial resistance mechanisms leading to multidrug-resistance (MDR) phenotypes , which further complicates clinical management [2]. Nationwide surveillance studies have found remarkably high rates of resistance among ocular bacteria, especially Gram-positives [3]. Ocular staphylococci isolates display the highest resistance levels among ocular bacteria, with a prevalence of methicillin resistance of around 35% for *S. aureus* [3]. Methicillin-resistant strains pose a major challenge for the management of eye infections as they are substantially more resistant to first-line antibiotics used for empirical treatment of ocular infections such as fluoroquinolones, macrolides, and aminoglycosides [3]. Molecular epidemiology studies have shown that the population structure of methicillin-resistant *S. aureus* (MRSA) causing ocular surface infections, especially keratitis, is dominated by epidemic MRSA lineages commonly associated with difficult-to-treat MDR infections in the hospital setting [4]. This substantially reduces the number of topical antibiotics that can be effectively deployed to treat these sight-threatening infections caused by MDR strains of MRSA.

Lincosamide antibiotics are among the limited armamentarium of antibiotics used to treat serious ocular infections. Lincosamides are mostly active against Gram-positive bacteria and are commonly used to treat human (mainly clindamycin) and animal infections [5]. Clindamycin has been mainly used to treat patients allergic to beta-lactams or anaerobic bacterial infections [5]. In ophthalmology, clindamycin has been extensively used in intravitreal injections to treat ocular toxoplasmosis, and sometimes for treatment and prophylaxis of bacterial endophthalmitis [6]. Clindamycin is also commonly given systemically to patients with orbital and periorbital infections [7]. Topical clindamycin solution has been shown to be safe and to penetrate the ocular tissues in animal models, and has been successfully used as an eyedrop or subconjunctival injection to treat ocular infections in humans [8]. Even though these advantages place clindamycin as a good candidate to treat ocular infections, it’s routine use is complicated by the emergence of antimicrobial resistance in ocular isolates [3], especially among ocular surface MRSA strains that often carry genes conferring resistance to lincosamides [4]. Resistance caused by methylation of the ribosomal target of the lincosamides by *erm* (erythromycin ribosome methylase) genes remains one of the most widespread mechanisms of resistance to lincosamides.

Iboxamycin (IBX) (Supplementary Fig. S1A) is a newly developed synthetic lincosamide with an exceptionally broad spectrum of antimicrobial activity against both Gram-positive and -negative bacteria [9]. As with clindamycin, IBX is a bacteriostatic antibiotic that inhibits protein synthesis by binding to the large ribosomal subunit (50S) of the bacterial ribosome, near the peptidyl transferase centre. IBX was found to be more potent than clindamycin when tested against a small collection of Gram-positive bacteria and was designed to overcome lincosamide resistance mediated by common widespread resistance mechanisms including 23S rRNA methylation by *erm* and *cfr* genes, conferring MLS_B_ (macrolides, lincosamides, and streptogramins B) and PhLOPS_A_ (phenicols, lincosamides, oxazolidinones, pleuromutilins, and streptogramins A) resistance phenotypes, respectively [9]. Recently, Wu et al. described the conformationally restricted antibiotic cresomycin (CRM), which further improves upon the antibacterial activity of IBX via a northern macrobicyclic thiolincosamine scaffold (Supplementary Fig. S1B) [10]. Both IBX and CRM were shown to have low *in vitro* cytotoxicity against multiple human cell lines, did not cause haemolysis of human erythrocytes, or did not exhibit mitochondrial toxicity. These molecules have also been tested in a mouse model of sepsis and were demonstrated to effectively treat animals infected with Gram-positive and -negative pathogens [9,10].

The *in vitro* and *in vivo* efficacy of IBX and CRM, as well as their tolerability profile, place these molecules as good candidates to be explored as potential novel therapies for ocular infections. Both of these antibiotics have been recently synthesised and not yet been explored in ophthalmology. Because of their activity against MDR MRSA isolates, including those with a MLS_B_ phenotype, which are becoming common causes of vision-threatening and recalcitrant ocular infections [3,11], these molecules could become important additions to the arsenal of therapies to treat increasingly resistant infections caused by MRSA. To begin exploring this, here we performed an *in vitro* activity study of IBX, CRM, and comparator antibiotics against a collection of genomically characterised clinical ocular MRSA isolates, including MDR strains carrying *erm* genes.

## 2. Material and methods

### 2.1. Bacterial isolates

A total of 85 ocular isolates were identified from patients presenting with eye infections at the Massachusetts Eye and Ear (MEE) Infirmary (Boston, MA, USA) from 2014 to 2017. From these, 50 isolates chosen to be representative of (i) the major ocular MRSA clones circulating in the USA and (ii) the clinical diversity of ocular MRSA infections [12] were included in this study. Methicillin resistance was confirmed phenotypically by oxacillin resistance and genotypically by the detection of the *mecA* gene.

Protocols for obtaining discarded isolates with waived informed consent were approved by the Mass General Brigham Institutional Review Board. Primary clinical specimens collected from multiple ocular infection sites were obtained by the attending ophthalmologist or resident following institutional guidelines and submitted to the clinical microbiology laboratory for processing. Bacterial identification was performed using the MicroScan WalkAway system (Beckman Coulter, Brea, CA, USA) following the manufacturer’s protocol. Isolates were stored at −80 °C in Microbank cryopreservative tubes (ProLab Diagnostics, Ontario, Canada). Frozen isolates were cultured on 5% sheep blood agar plates (BD Biosciences, San Jose, CA, USA) and incubated at 37 °C.

### 2.2. Antibiotic susceptibility testing

Isolates were tested for susceptibility by broth microdilution following the Clinical & Laboratory Standards Institute (CLSI) M07-A9 document. Quality assurance was performed by concurrently testing a CLSI-recommended strain (*S. aureus* ATCC29213). CLSI-approved interpretive break point criteria were also applied [13]. IBX and CRM were synthesised as previously described [9,10]. Minimum inhibitory concentration (MIC)_50_ and MIC_90_ were calculated using SPSS software (version 24, IBM, Armonk, NY, USA). As comparator antimicrobial agents, the following representative antibiotics from five different classes were tested, including: clindamycin (lincosamide); azithromycin (macrolide); tobramycin (aminoglycoside); moxifloxacin, levofloxacin, ofloxacin and besifloxacin (fluoroquinolones); and vancomycin (glycopeptide). MDR was defined as resistance to three or more classes of antibiotics.

### 2.3. Genome sequencing and assembly

Whole-genome sequencing was performed on the 50 isolates included in this study. Total DNA was purified from an overnight pure culture in 5 mL of Brain Heart Infusion broth using the DNeasy DNA extraction kit (Qiagen, Valencia, CA, USA). DNA quality was verified on a Bio-Tek Synergy 2 microplate reader (Winooski, VT, USA) prior to quantification using a Qubit fluorometer and dsDNA High-Sensitivity assay kit (Invitrogen, Carlsbad, CA, USA). Library preparation for Illumina sequencing was carried out using the Nextera XT DNA Library Preparation kit (Illumina, San Diego, CA, USA), according to the manufacturer’s specifications. The quality and quantity of each sample library was measured on a TapeStation instrument (Agilent Technologies, Santa Clara, CA, USA). The genomes were sequenced as 2 × 250 bp reads on an Illumina MiSeq sequencer, according to the manufacture’s specifications with a minimum depth of coverage of 30×. Sequence reads were assembled de *novo* using a CLC Genomics Workbench (CLC Bio, Cambridge, MA, USA).

### 2.4. Prediction of sequence types, clonal complexes, and antibiotic resistance genes

We used the Center for Genomic Epidemiology pipeline to obtain confirmation of species identification and sequence type (ST). Clonal complexes (CCs) were determined using MLST data and the goeBURST algorithm. The CARD (Comprehensive Antibiotic Resistance Database) algorithm was used to identify the pool of acquired antibiotic resistance genes. Staphylococcal Cassette Chromosome *mec* (SCC*mec*) was typed using SCC*mec*Finder v1.2.

## 3. Results

### 3.1. Source and characterisation of isolates

A total of 50 MRSA isolates recovered from eye infections were selected based on their MDR phenotypes and genotypic information regarding MLST types and the carriage of antimicrobial resistance genes. Isolates were recovered from periocular infections (*n* = 18, 36%), keratitis (*n* = 14, 28%), lacrimal system (*n* = 8, 16%), conjunctivitis (*n* = 5, 10%), and endophthalmitis (*n* = 5, 10%) (Table 1). Most MRSA isolates grouped within CC8 (*n* = 24), CC5 (*n* = 21), CC59 (*n* = 3), CC30 (*n* = 1), and ST6 (*n* = 1) (Table 1), which is representative of the common lineages that cause MRSA ocular infections [12]. The CC5 group was mostly comprised of isolates belonging to ST5 (*n* = 17), and the CC8 group was mostly comprised of isolates belonging to ST8 (*n* = 23) (Supplementary Table S1). SCC*mec* typing revealed that all MRSA CC8 (*n* = 24) were SCC*mec* type IV, a characteristic feature of lineage USA300 [14], whereas 76.2% (16/21) of MRSA CC5 had an SCC*mec* cassette type II, usually found in the MDR hospital-adapted lineage USA100 (Supplementary Table S1) [15].

### 3.2. *IBX and CRM are active against MRSA including strains harbouring* erm *genes*

To determine whether IBX and CRM are active against a collection of ocular MRSA with varying resistance towards the clinically used lincosamide, clindamycin, *in vitro* susceptibilities were determined by broth microdilution. IBX and CRM displayed high activity and potency against this collection of isolates and were substantially more potent than clindamycin (Table 2). CRM displayed the highest potency among these molecules (MIC_50_ 0.06 mg/L, MIC_90_ 0.5 mg/L, range: 0.03–0.5 mg/L). IBX had a comparable activity (MIC_50_ 0.06 mg/L, range: 0.015–2 mg/L) and a slightly lower (four-fold) potency (MIC_90_ 2 mg/L). Clindamycin had the lowest activity and potency (MIC_50_ 0.25 mg/L, MIC_90_ >16 mg/L, range: ≤0.12–>16 mg/L), with 35.3% of the isolates being classified as non-susceptible to this drug using CLSI breakpoints for categorisation.

As methylation of 23S rRNA due to *erm* genes leads to cross-resistance against macrolides, lincosamides, and streptogramins B, we were interested in testing the activity of IBX and CRM against a subgroup of isolates carrying *erm* genes. We used whole-genome sequence data to screen for the presence of *erm* genes using CARD and selected 25 isolates (out of 50) that were positive for either *ermA* (*n* = 18), *ermC* (*n* = 6), or *ermB* (*n* = 1) (Table 2). As expected, clindamycin lacked activity against isolates harbouring *erm* genes, showing a MIC_90_ 64-fold higher than its MIC_90_ in the *erm*-negative isolates (Table 2). However, IBX and CRM were highly potent against MRSA isolates harbouring *erm* genes *in vitro*, with an MIC_90_ of 2 and 0.5 mg/L, respectively (Table 2).

### 3.3. IBX and CRM display more potent activity against ocular MRSA compared with antibiotics commonly used to treat ocular infections

To determine the rates of antibiotic resistance and the drugs that would predict better treatment outcomes of bacterial ocular infections based on *in vitro* susceptibility profiles, we tested the *in vitro* susceptibilities of all isolates against IBX and CRM, and compared them with a panel of antibiotics commonly used in ophthalmology.

IBX and CRM antibiotics were substantially more potent *in vitro* against MRSA isolates than comparator drugs –they showed comparable activities with an MIC_50_ of 0.06 mg/L, which was the lowest MIC_50_ among all the antibiotics tested (Table 3). Overall, CRM displayed the highest *in vitro* potency (2–64-fold better; MIC_90_ 0.5 mg/L) compared with all other drugs tested. The *in vitro* potency of IBX (MIC_90_ 2 mg/L) was comparable to vancomycin and the newer fluoroquinolone besifloxacin (MIC_90_ 2 mg/L for both drugs) (Table 3).

Despite the community origins of our MRSA collection, we found a high prevalence of resistance to clinically important antibiotics that are often used for treatment of ocular infections, such as levofloxacin (60.8%) and azithromycin (90.2%) (Table 3). The later-generation fluoroquinolones (besifloxacin and moxifloxacin) had lower MIC_50_ and MIC_90_ overall compared with the earlier fluoroquinolones (ofloxacin and levofloxacin). Within the fluoroquinolones, besifloxacin showed the lowest MIC_50_ and MIC_90_, and ofloxacin showed the highest (Table 3). As shown in Supplementary Fig. S2, most of our MRSA isolates were concurrently resistant to other antibacterial drug classes, with 68.6% of MRSA being multidrug resistant (resistant to three or more antibiotic classes).

## 4. Discussion

In this study we have evaluated the *in vitro* activity of IBX, CRM, and comparator antibiotics against a collection of genomically characterised clinical ocular MRSA isolates, including MDR *erm*-expressing strains with MLS_B_ resistance phenotypes. *S. aureus* is a leading cause of ocular infections, including serious and sight-threatening diseases such as infectious keratitis and periocular cellulitis and abscess [1]. Nationwide multicentre surveillance studies show increasing and remarkably high antibiotic resistance levels among ocular pathogens, especially for MRSA [3,16]. Fluoroquinolones are the most commonly used antibiotics to prevent and treat these infections, but their overuse has resulted in the emergence of high levels of resistance to many molecules in the class, especially among MRSA isolates [3,17]. In our collection of isolates, approximately 60% of MRSA isolates were resistant to fluoroquinolones, a rate of resistance that is similar to what has been found in large-scale surveillance studies. For example, data generated by the Antibiotic Resistance Monitoring in Ocular Microorganisms (ARMOR) study have shown that approximately 70% of the ocular MRSA isolates (*n* = 765) collected from 2009 to 2018 in the USA were resistant to at least one fluoroquinolone antibiotic [3]. For other antibiotics, our results were also consistent with those from the ARMOR programme and other local studies, which found resistance to macrolides ranging from 92.9 to 96%, whereas aminoglycoside resistance was close to 40% for MRSA isolates [3,18].

Our results indicate that IBX and CRM represent a potential option for the treatment of ocular infections caused by MRSA isolates, including isolates that are now highly resistant to antimicrobials commonly used in the clinic. Both drugs were active against CC8 and CC5, two major and epidemic clonal complexes that are also common causes of MRSA infections in other body sites. CC8 is the most common community-acquired MRSA lineage in the USA, whereas the CC5 lineage has been found to be a leading cause of antibiotic-resistant hospital-associated MRSA infections in the USA [14,19]. IBX and CRM were found to be more potent than clindamycin against MRSA isolates. Moreover, we showed that IBX and CRM overcame lincosamide resistance mediated by *erm* genes, which are widespread antibiotic resistance determinants of clinical relevance [12,20]. *Erm* genes encode methyltransferase enzymes that dimethylate the N6 position of nucleotide A2058 of the 23S rRNA, which is part of the large (50S) ribosomal subunit. The overlapping binding sites of macrolides, lincosamides, and streptogramins B in 23S rRNA account for cross-resistance to the three classes of drugs. Thus, in the present *in vitro* study we demonstrated that IBX and CRM are active against a collection of ocular MRSA isolates, including MDR lineages frequently associated with keratitis, paving the way to *in vivo* and clinical studies to determine the positioning of such a therapy for human use.

In summary, this preclinical study demonstrated that synthetic lincosamides display potent *in vitro* activity against ocular MRSA, including MDR isolates, with potencies that are higher (CRM) or similar (IBX) to vancomycin and besifloxacin. The potencies of CRM and IBX are also higher than other comparator agents currently used as the standard of care for the treatment of ocular infections, especially when compared with older generations of fluoroquinolones, tobramycin, and azithromycin. Antimicrobial resistance is a global pandemic that poses a major threat to human health and is especially important in ophthalmology, as delays in effective treatment of eye infections and failures due to resistance can result in irreversible damage of key sensory functions. IBX and CRM are potential candidates that could be developed into improved local therapies for hard-to-treat and recalcitrant ocular infections caused by increasingly MDR MRSA isolates. Further *in vivo* studies are necessary to evaluate the eye tissue penetration and safety of these new drugs.

## Supplementary Material

1

2

## Figures and Tables

**Table 1:** Clonal complexes and source of isolation for ocular methicillin-resistant *Staphylococcus aureus* strains tested in this study.

Clonal complex (no. of isolates)	No. of isolates from:				
	Periocular infections^a^ *n* = 18	Keratitis *n* = 14	Lacrimal system *n* = 8	Conjunctivitis *n* = 5	Endophthalmitis *n* = 5
CC8 (24)	14	3	5	2	–
CC5 (21)	1	11	2	2	5
CC59 (3)	2	–	–	1	–
CC30 (1)	–	–	1	–	–
ST6 (1)	1	–	–	–	–

a Includes eyebrow abscess, orbital abscess and cellulitis, subperiosteal abscess, and preseptal cellulitis.

**Table 2:** Comparative antimicrobial activity of clindamycin, iboxamycin, and cresomycin tested against methicillin-resistant *Staphylococcus aureus* isolates causing ocular infections at the Massachusetts Eye and Ear Infirmary according to presence of *erm* genes.

Organism/compound	No. of isolates and cumulative % inhibited at MIC (mg/L) of:											MIC (mg/L)		
	0.015	0.03	0.06	0.12	0.25		0.5		1	2	>16	MIC_50_	MIC_90_	
**MRSA (*n* = 50)**														
Iboxamycin	1 (2.0)	22 (46.0)	8 (60.0)	1 (62.0)			2 (68.0)		4 (76.0)	12 (100.0)		0.06	2	
Cresomycin		14 (28.0)	18 (64.0)	2 (68.0)	9 (86.0)		7 (100.0)					0.06	0.5	
Clindamycin				8 (16.0)^b^	24 (64.0)				1 (66)		17 (100)	0.25	> 16	
***erm*^a^-positive isolates (*n* = 25)**														
Iboxamycin		5 (20.0)	2 (28.0)	1 (32.0)			1 (36.0)		4 (52.0)	12 (100.0)		1	2	
Cresomycin		2 (8.0)	6 (32,0)	2 (40.0)	8 (72.0)		7 (100.0)					0.25	0.5	
Clindamycin				3 (12.0)	6 (36.0)				1 (40.0)		15 (100)	> 16	> 16	
***erm*-negative isolates (*n* = 25)**														
Iboxamycin	1 (4.0)	17 (72.0)	6 (96.0)				1 (100)					0.03	0.06	
Cresomycin		12 (48.0)	12 (96.0)		1 (100)							0.06	0.06	
Clindamycin				5 (20.0)	18 (92.0)						2 (100)	0.25	0.25	

a *ermA n* = 18, *ermC n* = 6, *ermB n* = 1.

b Lowest concentration tested for clindamycin.

**Table 3:** Comparative antimicrobial activity of iboxamycin, cresomycin, and comparator agents tested against MRSA isolates causing ocular infections at the Massachusetts Eye and Ear Infirmary.

Organism/resistance group (no. tested/antimicrobial agent)	MIC (mg/L)			CLSI, %S/%NS
	MIC_50_	MIC_90_	Range	
**MRSA (*n* = 50)**				
Iboxamycin	0.06	2	0.015–2	
Cresomycin	0.06	0.5	0.03–0.5	
Clindamycin	0.25	>16	≤0.012–>16	64.7/35.3
Besifloxacin	≤0.25	2	≤0.25–8	
Moxifloxacin	1	8	≤0.25–>32	45.1/54.9
Levofloxacin	4	>32	≤0.25–>32	39.2/60.8
Ofloxacin	16	>32	≤0.25–>32	39.2/60.8
Tobramycin	0.5	>16	≤0.012–>16	68.6/31.4
Azithromycin	>16	>16	≤0.012–>16	7.8/92.2
Vancomycin	1	2	0.25–2	100.0/0.0

**NS**: Non-susceptible; **S**: Susceptible.
