# Supplementary material for: Synthetic lincosamides iboxamycin and cresomycin are active against ocular multidrug-resistant methicillin-resistant Staphylococcus aureus carrying erm genes
Source: J Glob Antimicrob Resist. Author manuscript; Available in PMC 2025 Jan 27. (PMC11771510; doi:10.1016/j.jgar.2024.09.001)
Supplement: 1 [file NIHMS2044005-supplement-1.docx]

**Supplementary figures**

**Figure S1. A.** Chemical structure of iboxamycin. **B.** Chemical structure of conformationally restricted macrobicyclic antibiotic cresomycin.

**Figure S2.** Concurrent resistance to antibiotics.

Multidrug resistance is defined by resistance to 3 or more antibiotic classes.
